# Curcumin diethyl disuccinate, a prodrug of curcumin, enhances anti-proliferative effect of curcumin against HepG2 cells via apoptosis induction

**DOI:** 10.1038/s41598-019-48124-1

**Published:** 2019-08-12

**Authors:** Chawanphat Muangnoi, Pahweenvaj Ratnatilaka Na Bhuket, Ponsiree Jithavech, Wiwat Supasena, Luminita Paraoan, Suthiluk Patumraj, Pornchai Rojsitthisak

**Affiliations:** 1 Pharmaceutical Chemistry and Natural Products Program, Faculty of Pharmaceutical Sciences, Chulalongkorn University, Bangkok, 10330 Thailand; 2 Natural Products for Ageing and Chronic Diseases Research Unit, Chulalongkorn University, Bangkok, 10330 Thailand; 3 Institute of Ageing and Chronic Disease, University of Liverpool, Liverpool, UK; 4 Center of Excellence for Microcirculation, Department of Physiology, Faculty of Medicine, Chulalongkorn University, Bangkok, 10300 Thailand; 5 Department of Food and Pharmaceutical Chemistry, Faculty of Pharmaceutical Sciences, Chulalongkorn University, Bangkok, 10330 Thailand

**Keywords:** Cancer models, Mechanisms of disease

## Abstract

Curcumin (Cur) has been reported to have anti-hepatocellular carcinoma activity but its poor oral bioavailability limits its further development as a chemotherapeutic agent. We synthesized previously a succinate ester prodrug of Cur, curcumin diethyl disuccinate (CurDD) with better chemical stability in a buffer solution pH 7.4. Here, we further investigated and compared the cellular transport and anti-proliferative activity against HepG2 cells of CurDD and Cur. Transport of CurDD across the Caco-2 monolayers provided a significantly higher amount of the bioavailable fraction (BF) of Cur with better cytotoxicity against HepG2 cells compared to that of Cur (*p* < 0.05). Flow cytometric analysis showed that the BF of CurDD shifted the cell fate to early and late apoptosis to a higher extent than that of Cur. The Western blot analysis revealed that CurDD increased Bax protein expression, downregulated Bcl-2 protein, activated caspase-3 and -9 and increased LC3-II protein level in HepG2 cells. Flow cytometric and immunoblotting results suggest that CurDD can induce HepG2 cell death via an apoptotic pathway. We suggest that CurDD can overcome the limitations of Cur in terms of cellular transport with a potential for further extensive *in vitro* and *in vivo* studies of anti-hepatocellular carcinoma effects.

## Introduction

**Figure 1:**
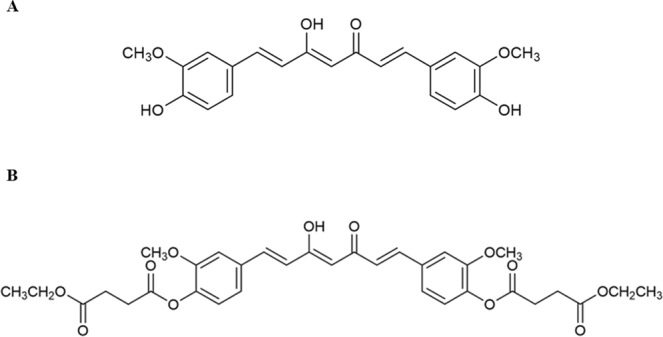
Chemical structures of (**A**) curcumin (Cur) and (**B**) curcumin diethyl disuccinate (CurDD).

Curcumin (Cur, Fig. 1A) is a major bioactive curcuminoid found in turmeric (*Curcuma Longa* L.). Several pharmacological activities of Cur have been reported, including anti-inflammation^1,2^, anti-oxidant^3^, neuroprotection^4^ and anti-angiogenesis^5^. It has been reported as the nutraceutical for chronic diseases including anticancer activity against several cancer cells such as leukemia, lung cancer, brain cancer, breast cancer and colon cancer^6–11^. Cur inhibits the growth of cancer cells by increasing Bcl-2 Associated X (Bax) protein expression and suppressing B-cell lymphoma 2 (Bcl-2) protein expression^12–14^, which in turn activate caspase-3 and -9 and induce apoptosis. Cur has also been reported to inhibit cancer cell growth through the activation of autophagic signaling pathways^15,16^. Kim *et al*. showed that Cur induced autophagic cell death in oral cancer cells as demonstrated by the increase in the lipidated microtubule-associated protein 1A/1B-light chain 3 (LC3-II) protein level, which is an indicator of autophagosome formation^17^. Jia *et al*. reported that Cur could induced apoptotic and autophagic cell death in K562 leukemia cells by increasing caspase-3 and LC3-II protein levels while the Bcl-2 protein expression was decreased^18^. Despite the fact that Cur has prominent anticancer effects, its application as a chemotherapeutic agent is limited by the poor oral bioavailability due to the chemical and metabolic instability along the gastrointestinal (GI) tract^19,20^.

Several strategies, including formulation and prodrug approaches, have been used to overcome the chemical and metabolic instability of Cur for the enhancement of its oral bioavailability^21–23^. Various formulations have been prepared by physical incorporation of Cur in micelles, niosomes, liposomes, phospholipids, nanoparticles for stability enhancement^21,23^. Prodrug approach has been employed to improve stability and bioavailability of several pharmaceuticals and phytochemicals. Previously, we have developed several Cur ester prodrugs by conjugating Cur to dicarboxylic acids and polymers^24^. Curcumin diethyl disuccincate (CurDD, Fig. 1B) is one of our Cur ester prodrugs with improved chemical stability in phosphate buffer pH 7.4 and increased anti-proliferative activity against colon and breast cancer cell lines^25,26^. Pharmacokinetic studies of CurDD showed that CurDD had superior tissue distribution in comparison with Cur and improved an area under the plasma concentration versus time curve of Cur after oral and intravenous administration^27,28^. It also possesses a promising analgesic activity in the peripheral and central anti-nociceptive models in mice^29^. Therefore, CurDD has a potential to be developed as an anti-nociceptive and anticancer candidate.

Caco-2 cell line, a human colon epithelial cancer cell line, has been widely used as an *in vitro* model for studying compound absorption and intestinal metabolism for drugs intended for oral administration^30,31^. After being cultured for 3 weeks, these cells can differentiate such that morphologically resemble the enterocytes of the small intestine.The resulting monolayers have tight junctions, microvilli and brush-border characteristics at the apical side, and various enzymes for phase I and phase II metabolism and transport proteins^32,33^. This *in vitro* model has been used to investigate the permeation and to predict the intestinal absorption as well as gut metabolism of several compounds e.g. pivampicillin, cefcapene pivoxil hydrochloride, and monocarbonyl Cur analogues^34,35^.

Cur was found to be converted to several reduced and conjugated metabolites by Caco-2 cells^36^. CurDD is an ester prodrug of Cur that requires bioconversion before exhibiting pharmacological activities. In this study, we exposed Cur and CurDD to Caco-2 monolayers for the bioconversion during cellular transport. Then, we investigated and compared the anti-proliferative effect and mechanism against HepG2 cells of Cur and CurDD after being transported across the Caco-2 monolayers. We show that CurDD could improve the transport of Cur across the Caco-2 monolayers and anti-proliferative activity against HepG2 cells of Cur.

## Materials and methods

### Chemicals and materials

Cur ((1E,6E)-1,7-bis(4-Hydroxy-3-methoxyphenyl)hepta-1,6-diene-3,5-dione, MW 368.4, purity >98% by high-performance liquid chromatography (HPLC)) and CurDD (4,4′-((1E,6E)-3,5-Dioxohepta-1,6-diene-1,7-diyl)bis(2-methoxy-1,4-phenylene)diethyl disuccinate, MW 624.6, purity >98% by HPLC) were synthesized as previously described and characterized by proton nuclear magnetic resonance (^1^H-NMR)^25^. 3-[4,5-dimethyltiazol-2-yl]-2,5-diphenyl-tetrazolium bromide (MTT), dimethyl sulfoxide (DMSO) and other chemicals were purchased from Sigma-Aldrich (St. Louis, MO, USA). Dulbecco’s modified Eagle’s medium (DMEM), L-glutamine, nonessential amino acids, penicillin and streptomycin, and fungizone were obtained from Invitrogen (Grand Island, NY, USA). Fetal bovine serum (FBS) was purchased from PAA Laboratories (Haidmannweg, Austria). Authenticated human colorectal adenocarcinoma (Caco-2) and human hepatocellular carcinoma (HepG2) cells were obtained from the American Type Culture Collection (ATCC, Rockville, MD, USA). Primary antibodies against Bax, Bcl-2, LC3B and β-actin were purchased from Cell Signaling Technology (Danvers, MA, USA). The secondary antibodies were purchased from Cell Signaling Technology (Danvers, MA, USA).

### Evaluation of cytotoxicity of Cur and CurDD against Caco-2 cells

Cytotoxicity against Caco-2 cells was evaluated prior to the investigation of cellular transport of Cur and CurDD. Cells were seeded in 96-well plates at a density of 1 × 10^4^ cells/well and incubated at 37 °C in a humidified atmosphere of 95% air:5% CO_2_ for 24 h. Cells were divided into three groups: control group (DMSO), Cur group and CurDD group. After incubation, cells were washed with serum free medium and then 200 µL of serum free medium were added in each well prior to an addition of 2 µL of DMSO (0.5% DMSO at final concentration) or samples (Cur and CurDD) at various concentrations (final concentration at 0.1–20 µM). Treated cells were incubated for 4 h at 37 °C in humidified atmosphere of 95% air/5%CO_2_. After incubation, cell viability of treated cells was determined by MTT assay. Experiments were performed in four replicates. Results are presented as % cell viability in comparison with the control.

### Evaluation of cellular transport of Cur and CurDD

**Figure 2:**
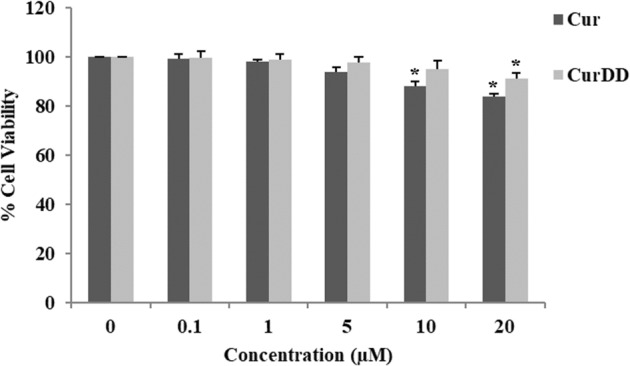
Cell viability of Caco-2 cells incubated with Cur or CurDD at various concentrations (0.1–20 µM) for 4 h. Data presented is mean ± SD values of four replicates *p < 0.05 indicates significant differences from the control group.

Caco-2 cells (passage 25–35) were cultured in a complete medium (DMEM supplemented with 15% heat inactivated FBS (v/v), 1% L-glutamine (v/v), 1% nonessential amino acids (v/v), 1% penicillin and streptomycin (v/v) and 0.2% fungizone). Caco-2 cells were seeded in trans-well inserts of 6-well plates (ThinCerts^TM^-TC Einsatze, Greiner Bio-one, Switzerland) at a density of 2.5 × 10^4^ cells/2 mL of complete medium /well in an apical compartment. A basolateral compartment was added with 2 mL of phenol red-free DMEM. Cells were incubated at 37 °C in a humidified atmosphere of 95% air/5% CO_2_. The serum content of the complete medium was decreased to 7.5% once the cultures reached confluency. The complete medium was changed every other day. At 21–24 days after confluence with the trans epithelial electrical resistance (TEER) more than 500 Ω/cm^2^, differentiated monolayers were washed with serum free medium before adding 2 mL of serum free medium containing Cur or CurDD at 5 µM (non-toxic concentration, Fig. 2) in the apical compartment. The plates were returned to an incubator at 37 °C. The medium in the basolateral compartment of treated samples was collected at 15, 30, 60, 120, and 240 min. The fluids collected at different time intervals were designated as bioavailable fractions (BF). Experiments were performed in four replicates. The collected BF were blanked with nitrogen gas and stored at −80 °C for further experiments.

### Extraction of Cur from BF for HPLC analysis

Briefly, a 2 mL of each BF was transferred into a 15-mL centrifuge tube. Ethyl acetate was added to the mixture at the ratio of 1:1, sonicated for 10 min, vortexed for 2 min and centrifuged (ROTINA 380R, Germany) at 4,000 *g* for 10 min. The organic layer was collected into a 10 mL vial. The extraction was repeated twice and the organic layers were combined and dried under nitrogen gas blowing. The residue was reconstituted with a mobile phase prior to HPLC analysis.

### Chromatographic conditions

The amounts of Cur were determined by HPLC analysis using a Dionex® ultimate 3000 system equipped with two pumps, autosampler, diode-array detector, and Chromeleon Dionex 1996–2006 version 6.80 SR15 Build 4656 (243203) software. The separation was carried out on a reversed-phase HALO C8 column (4.6 × 50 mm, 2.7 µm). A mobile phase comprised of acetonitrile and 2% acetic acid (38:62, v/v) was delivered isocratically at a flow rate of 1.0 mL/min. The detection wavelength was set at 400 nm.

### Evaluation of anti-proliferative effect of BF of Cur and CurDD

HepG2 cells (passage 50–60) were cultured in a complete medium (DMEM supplemented with 10% FBS (v/v) and 1% penicillin and streptomycin (v/v)) and seeded at a density of 1 × 10^4^ cells/well in 96-well plates for cytotoxicity test and at a density of 1.0 × 10^5^ cells/well in 6-well plates for investigation of apoptosis induction. The cells were incubated at 37 °C in a humidified atmosphere of 95% air/5% CO_2_ for 24 h. Cells were divided into three groups: control group (treated with the serum free medium), BF of Cur treated group and BF of CurDD treated group. The preparation of BF of Cur and CurDD were similar to that described in section cellular transport at 4 h.

### Cell viability test

HepG2 cells in the 96-well plates were washed with serum free medium before adding 200 µL of sample (BF of Cur or CurDD) and were incubated for 24 h. Then, the viability of treated cells was determined by MTT assay using a microplate reader (CLARIOstar, BMG LABTECH, Germany). Experiments were performed in four replicates. DMSO (0.5% DMSO final concentration) was used as a control. Results are presented as % cell viability in comparison with the control.

### Flow cytometric analysis

The cells in the 6-well plate were washed with serum free medium, then 2 mL of samples (BF of Cur or CurDD) were added and incubated for 24 h. Then, the cells were trypsinized by adding 1 mL of trypsin and incubated at 37 °C for 5 min. Following addition of 1 mL of 10% FBS in DMEM, cells were transferred into a 15-mL centrifuge tube and centrifuged at 800 *g* (ROTINA 380R, Germany) at 37 °C for 5 min. Cell pellets were resuspended in serum free medium and stained with Annexin V (Guava^®^ Nexin Reagent, MerckMillipore, Germany) at the ratio 1:1 before flow cytometric analysis (Guava^®^easyCyte Flow Cytometers, MerckMillipore, Germany).

### Determination of caspase-3 and -9 activities

The method for determining caspase activation was performed as previously described with some modifications^37^. Briefly, HepG2 cells were seeded at 1 × 10^6^ cells/mL in 6-well culture plates. After incubation for 24 h, the cells were treated with the BF of Cur or CurDD for 24 h. The treated cells were lysed in a hypotonic buffer (20 mM Tris-HCl pH 7.5, 1 mM ethylenediaminetetraacetic acid, 100 µM phenylmethanesulfonyl fluoride, 2 µg/mL aprotinin, pepstatin, and leupeptin). The supernatant was collected and incubated at 37 °C for 1 h with 100 µM *N-*acetyl-Asp-Glu-Val-Asp *p-*nitroanilide or *N*-acetyl-Leu-Glu-His-Asp *p*-nitroanilide as a specific substrate of caspase-3 and 9, respectively. The absorbance at 405 nm was measured using the microplate reader (CLARIOstar, BMG LABTECH, Germany).

### Western blot analysis

HepG2 cells were seeded at 1 × 10^6^ cells/mL in a 6-well culture plate. After 24 h for incubation, the cells were washed with the serum free medium (free phenol red) and treated with the BF of Cur or CurDD for 24 h. The treated cells in the 6-well plate were resuspended in an ice-cold lysis buffer for 30 min at 4 °C and centrifuged at 13,500 g at 4 °C for 5 min. Equal amounts (40 µg) of each protein sample were applied to 10% sodium dodecyl sulfate polyacrylamide gels and subjected to electrophoresis. Then, the protein samples were transferred to a pure nitrocellulose membrane (Amersham™Protran®, Sigma Aldrich), and blocked with 5% dry milk. The membrane was incubated with primary antibodies against cleaved caspase-3 (1:1000), cleaved caspase-9 (1:1000) Bax (1:1000), Bcl-2 (1:1000), LC3B (1:1000) or β-actin (1:5000) at 4 °C overnight. Then, membranes were washed with Tris buffered saline with Tween 20 and incubated with species-specific horseradish peroxidase conjugated secondary antibody reacted with Super Signal solution (Endogen Inc, Rockford, IL, USA) for 2 min. The membrane was exposed to an X-ray film, stripped off the bound antibody and re-probed with anti-β actin antibody to confirm the equal loading of protein. The density of target bands was quantified with the Image J program (downloaded from http://rsb.info.nih.gov/ij/). Results were expressed as a relative ratio of band intensity of the target proteins and β-actin.

### Statistical analysis

All experiments were performed at least in four replicates unless indicated otherwise. Statistical analysis was performed using SPSS version 13.0 (SPSS Inc., USA). Data are presented as mean ± standard deviation (SD). Means were compared by one-way analysis of variance (ANOVA) with Scheffe and Duncan’s post hoc test. Differences were considered significantly at *p* < 0.05.

## Results

### Assessment of cytotoxicity of Cur and CurDD in Caco-2 cells

The evaluation of cytotoxicity of Cur and CurDD was performed on Caco-2 cells prior to the cellular transport study. The results, presented as % cell viability in Fig. 2, indicated that the maximum concentrations of Cur and CurDD at 5 and 10 µM, respectively, had no difference in cell viability of Caco-2 cells compared with the control group. Therefore, the concentration of Cur and CurDD used for subsequent studies was 5 µM.

### Investigation of cellular transport of free Cur resulting from Cur and CurDD

**Figure 3:**
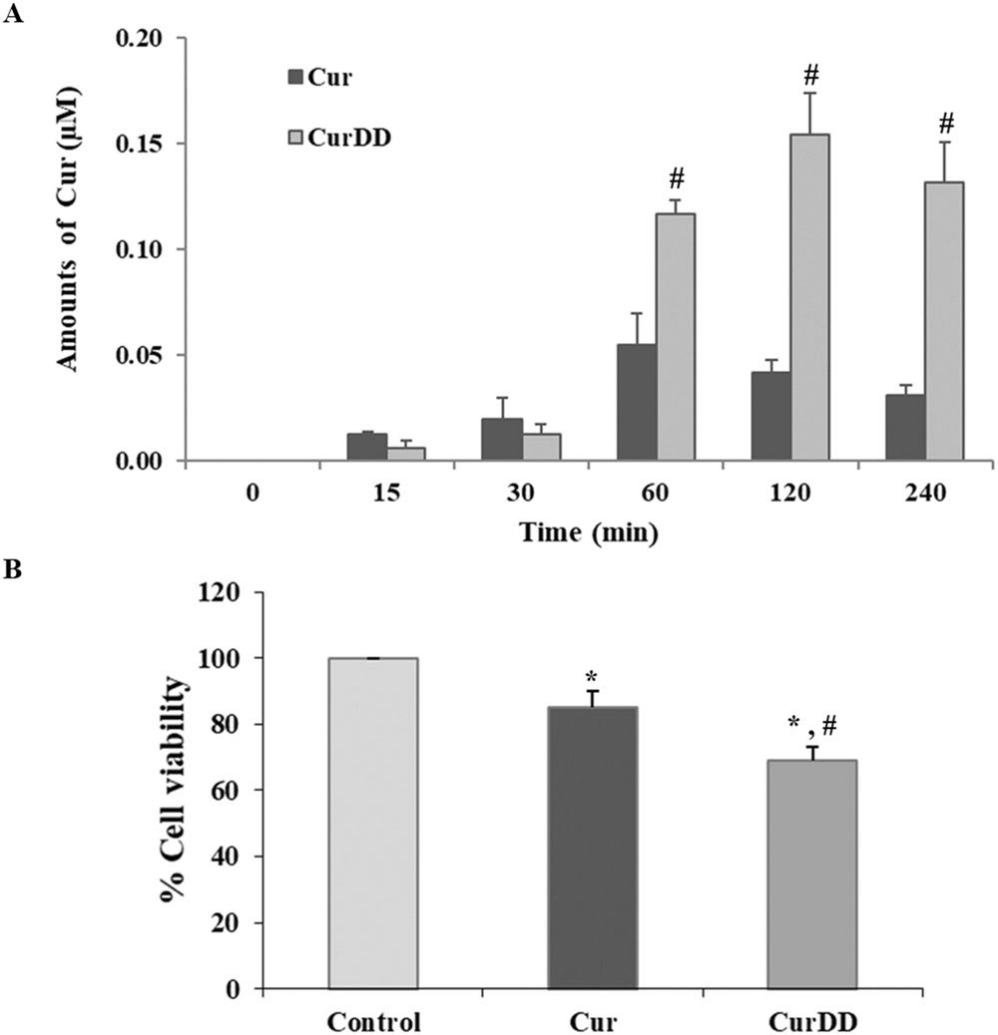
(**A**) Amounts of free Cur from cellular transport of Caco-2 cells (bioavailable fraction) derived from 5 µM of Cur or CurDD at different time intervals. (**B**) Cell viability of HepG2 cells incubated with the respective bioavailable fraction of Cur or CurDD for 24 h. Data presented is mean ± SD values of four replications. *p < 0.05 indicates significant differences from the control group; ^#^*p* < 0.05 indicates significant differences from the Cur group.

The time profiles of Cur derived from Cur or CurDD permeating across the Caco-2 cells are depicted in Fig. 3A. The amount of Cur from the BF of the Cur-treated group at 15 min was 0.013 µM. The amount of Cur increased over time to the maximum of 0.055 µM at 60 min and decreased steadily after that. The remaining amount of Cur at 4 h in the BF of the Cur-treated group was only 0.031 µM. Although CurDD showed a similar transport profile to that of Cur, the amount of free Cur found in the BF of CurDD was significantly different. Thus, at 15 min, the amount of free Cur was 0.006 which was about 2 times lower than in the Cur-treated group. The amount of Cur in the BF of CurDD reached the maximum of 0.154 µM at 120 min. This maximum amount of Cur in the BF of CurDD-treated group was 3 times higher than the maximum amount of Cur resulting from the BF of Cur. The remaining amount of free Cur at 4 h in the BF of CurDD-treated group was 0.132 µM, which was 4 times higher than the remaining amount of free Cur at 4 h in the BF of Cur-treated group.

### Anti-proliferative effect of the BF of Cur and CurDD against HepG2 cells

**Figure 4:**
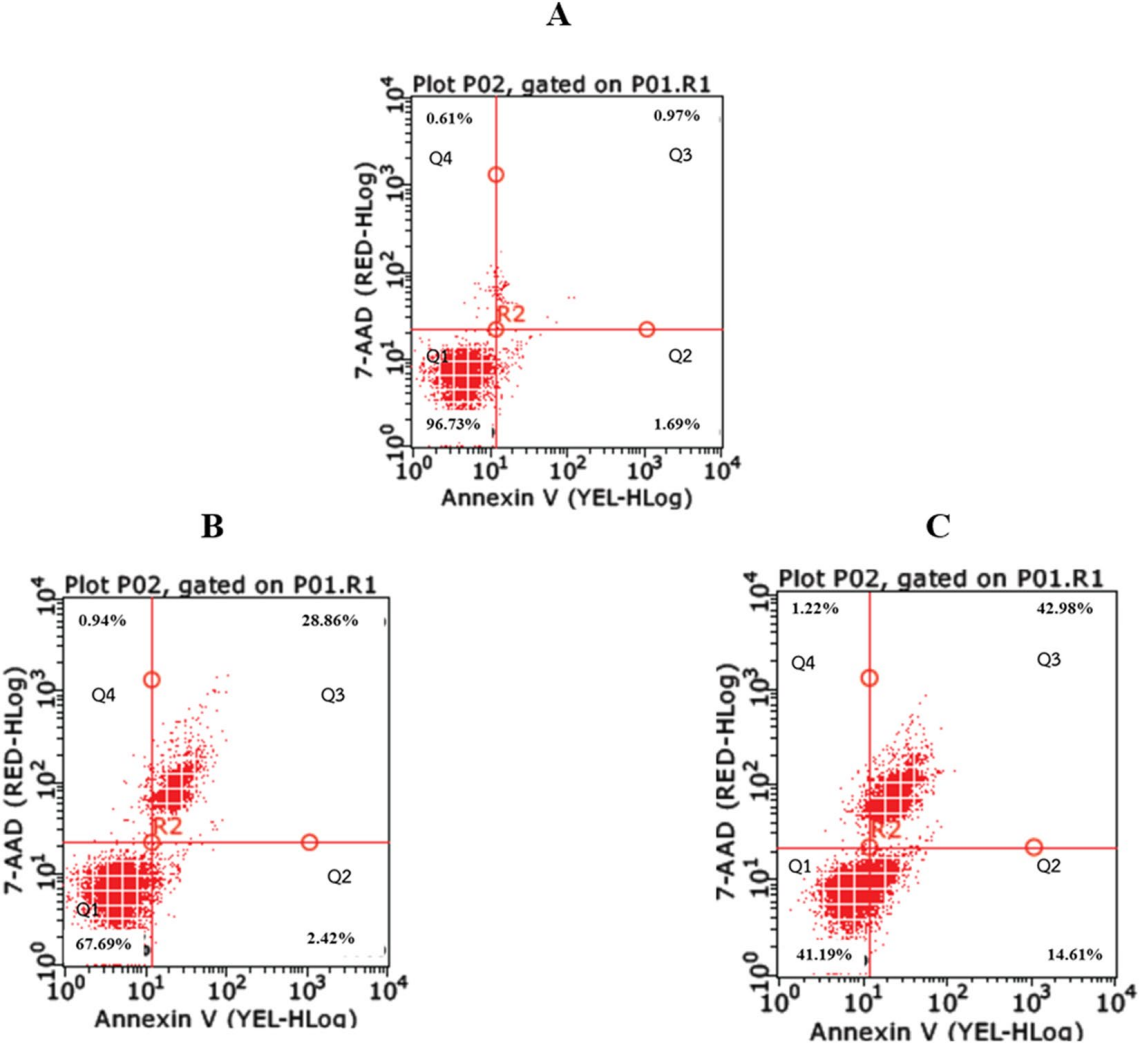
Flow cytometry results of HepG2 cells after treating with bioavailable fractions of Cur or CurDD. (**A**) Control group. (**B**) Treated with bioavailable fraction of Cur. (**C**) Treated with bioavailable fraction of CurDD. Q1 = Live cells, Q2 = Early apoptosis, Q3 = Late apoptosis and Q4 = Necrosis.

The anti-proliferative effects of the BF of Cur and CurDD was determined based on cytotoxicity tests and apoptosis assays. The cytotoxic effects of the BF of Cur and CurDD against HepG2 cells are presented as % cell viability in Fig. 3B. The % cell viability values of HepG2 cells after treating with the BF of Cur and CurDD were 85 and 69%, respectively. The outcomes of the apoptotic induction by the BF of Cur and CurDD in HepG2 cells are shown in Fig. 4, indicating that HepG2 underwent early and late apoptosis. The % cell population in the early and late apoptosis after incubating HepG2 cells with the BF of Cur for 24 h was found at 2.82 and 28.86%, respectively. The BF of CurDD also induced apoptosis in HepG2 cells with the % cell population in the early and late of apoptosis of 14.61% and 42.98%, respectively. These results indicated that the BF of CurDD had anti-proliferation effect on HepG2 cells by apoptosis induction in HepG2 cells at significantly higher levels than that of Cur. The amount of Cur in the BF was subsequently determined to confirm that the difference in the anti-hepatocellular activity between the BF of Cur and CurDD resulted from the different amount of Cur being actively transported.

### Effects of the BF of Cur and CurDD on caspase-3 and -9 activities and expression

**Figure 5:**
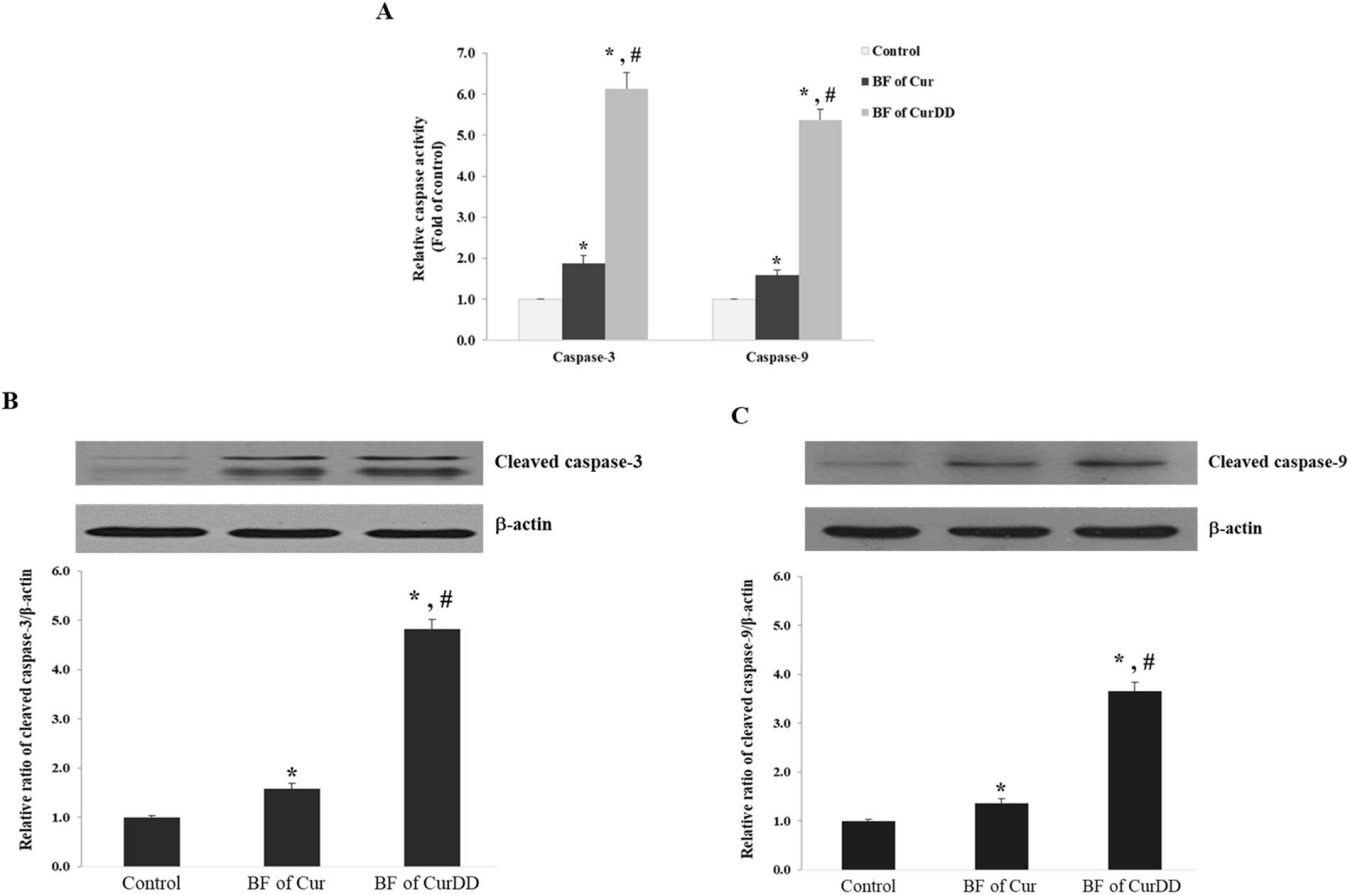
Effect of bioavailable fraction (BF) of Cur and CurDD on (**A**) caspase-3 and -9 activities (**B**) the expression of cleaved caspase-3 and (**C**) cleaved caspase-9 expression. The BF from the group without compound treatment was used as a control. Values represent means ± SD of four replicates. *p < 0.05 indicates significant differences from the control group; ^#^*p* < 0.05 indicates significant differences from the Cur group.

The profiles of caspase-3 and -9 activities and expression after incubating HepG2 cells with the BF of Cur and CurDD are shown in Fig. 5. Incubation of HepG2 cells with the BF of Cur increased caspase-3 and -9 activities by 1.8 and 1.5 times, respectively (Fig. 5A). The BF of CurDD also increased the caspase-3 and -9 activities by 6.1 and 5.3 times, respectively (Fig. 5A). To confirm the increase of caspase-3 and -9 activities occurred from the induction of protein expression, the expression of the cleaved caspase-3 and -9 was determined by Western blot. We found that the treatment HepG2 cells with the BF of Cur increased the cleaved caspase-3 and -9 expression by 1.5 and 1.3 times, respectively (Fig. 5B,C). The BF of CurDD also increased the caspase-3 and -9 activities by 4.8 and 3.7 times, respectively (Fig. 5B,C). The apoptosis induction effect of the BF of CurDD was thus significantly higher than that of Cur in relation to both induced caspase-3 and -9 activities and expression.

### Effects of the BF of Cur and CurDD on Bax and Bcl-2 protein expression

**Figure 6:**
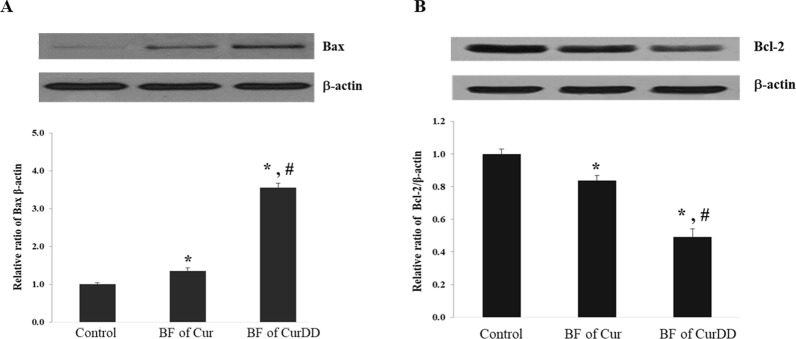
Effect of bioavailable fraction (BF) of Cur and CurDD on (**A**) Bax and (**B**) Bcl-2 expression. The BF from the group without compound treatment was used as a control. Values represent means ± SD of four replicates. *p < 0.05 indicates significant differences from the control group; ^#^*p* < 0.05 indicates significant differences from the Cur group.

Exposure of HepG2 cells to the BF of Cur and CurDD increased Bax (pro-apoptotic) protein by 13 and 40%, respectively, while decreasing Bcl-2 (anti-apoptotic) protein expression protein by 16 and 50% times, respectively (Fig. 6A,B). These results indicated that treatment of HepG2 cells with the BF of CurDD increased and respectively decreased the expression of Bax and Bcl-2 proteins, to a higher extent than in the case of the BF of Cur.

### Effects of the BF of Cur and CurDD on LC3-II protein level

**Figure 7:**
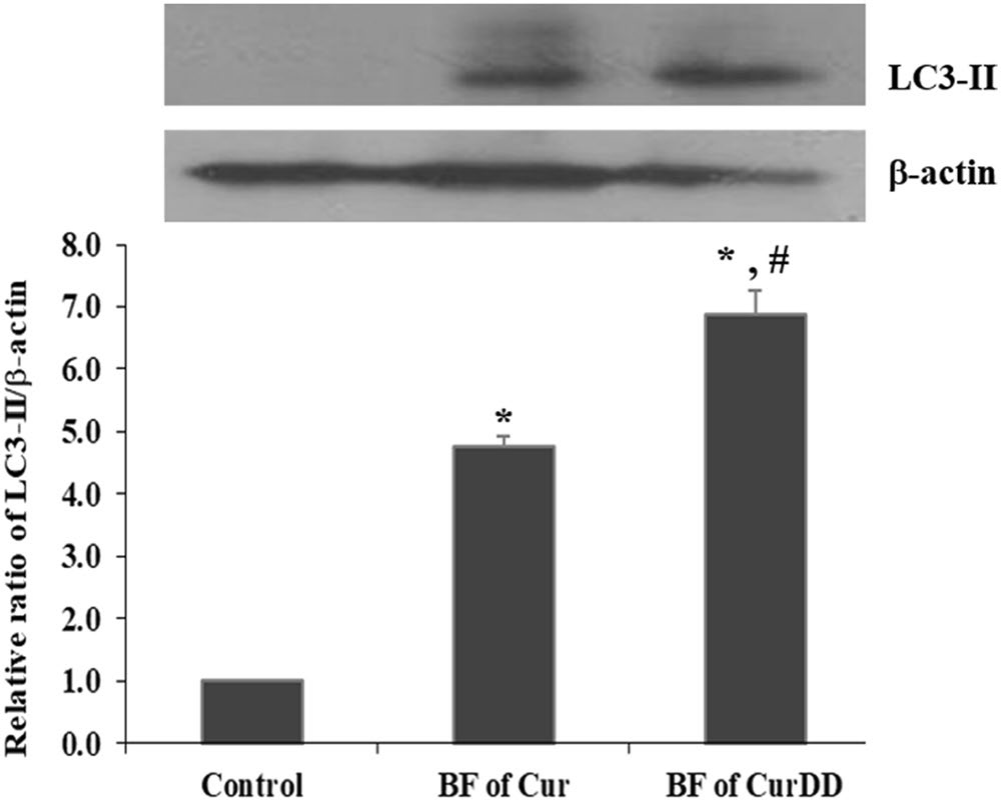
Effect of bioavailable fraction (BF) of Cur and CurDD on the LC3-II level. The BF from the group without compound treatment was used as a control. Values represent means ± SD of four replicates. *p < 0.05 indicates significant differences from the control group; ^#^*p* < 0.05 indicates significant differences from the Cur group.

Figure 7 shows that the incubation of HepG2 cells with the BF of Cur and CurDD significantly increased the level of LC3-II by 4.8 and 6.9 times, respectively (*p* < 0.05). Thus, the BF of CurDD increased the level of LC3-II significantly higher than the BF of Cur (*p* < 0.05).

## Discussion

The aims of the present study were to determine the anti-proliferative effects and mechanisms of CurDD, a succinate ester prodrug of Cur *in vitro* in comparison with Cur. Cur and CurDD were subjected to the bioconversion by the Caco-2 monolayers before being investigated for the anti-proliferative effects. We found that, after passing across the Caco-2 monolayers, which served as an *in vitro* intestinal epithelium lining, the BF of CurDD exhibited better cytotoxic effects against HepG2 cells compared to those of Cur. This could be due to the higher concentration of Cur found in the BF of CurDD compared to that resulting from administration of unmodified Cur. The differences in anti-hepatocellular carcinoma activities and the amounts of Cur found in the BF of Cur and CurDD might relate to the biotransformation in Caco-2 cells. Cur has been shown that it is extensively metabolized to several reduced and conjugated metabolites while being transported across Caco-2 cells^35,36^. Hence, the low amounts of Cur found after permeating across the Caco-2 monolayers detected in our study. On the other hand, CurDD is subjected to hydrolysis by intracellular esterase(s) and releases Cur. The presence of two ethyl succinyl groups might allow the released Cur to circumvent the reductive and conjugative metabolic reactions in Caco-2 cells, resulting in the significantly higher amounts of Cur found in the BF of CurDD.

Of note is the fact that the concentration of Cur in both BF is very low (less than 1 μM) but despite this it could exert the significant cytotoxicity against HepG2 cells compared to the non-treated control (*p* < 0.05). The observed cytotoxicity may be due to the metabolites of Cur resulting during cellular transport. Possible metabolites of Cur include reductive metabolites tetrahydroCur, hexahydroCur and octahydroCur, as well as their sulfate and glucuronide conjugates. These metabolites were previously reported to possess various biological activities with different extent of their potency^36^. We therefore suggest that, in addition to Cur, the cytotoxic effect might be derived from the Cur metabolites generated during the transport across Caco-2 monolayers.

Cur has been shown to induce apoptosis in various cancer cells like leukemia, breast, lung, colon and hepatocellular carcinomas^12,14^. In this study, we investigated and compared the patterns of apoptosis induction of Cur and CurDD using the BF of Cur and CurDD. The flow cytometric analysis showed that the BF of CurDD shifted HepG2 cells to early and late apoptosis to a higher extent than that of Cur. These findings could be explained by an increase in caspase activities and an alteration of the apoptotic protein expression. In the apoptosis pathway, there are 14 caspases identified in mammals but caspase-3 and -9 are demonstrated to be the most essential caspases for apoptosis^38,39^. Caspase-3, as an effector caspase, can cleave and suppress certain vital cellular proteins by proteolysis, while caspase-9, as an initiator caspase, can initiate a cascade of caspase activity by processing and activating effector caspases^38–40^. The BF of CurDD induced a 3-time enhancement of the caspase-3 and -9 activities and expression in comparison with the BF of Cur. Bax and Bcl-2 proteins are apoptotic and anti-apoptotic proteins, respectively, which are regulated by caspase-3 and -9^41,42^. The substantial increase in Bax protein expression and down regulation of Bcl-2 protein after treating HepG2 cells with the BF of CurDD were in agreement with the change in caspase activities and expression. These data indicated that the BF from both Cur and CurDD exhibited the anti-proliferative effect against HepG2 cells by inducing apoptosis in HepG2, with the BF of CurDD having greater effects.

In this study, the upregulation of the LC3-II level was observed after 24 h of incubation, suggesting that the BF of Cur and CurDD may induce autophagy in HepG2 cells. The increase in LC3 level can be related to either the enhancement of autophagosome formation or the impairment of autophagic degradation^43^. According to the flow cytometric and immunoblot results, we postulate that the BF of Cur and CurDD may initially induce autophagy in the HepG2 cells followed by apoptosis induction. Previously, Zhou *et al*. reported that a Cur analog, EF25-(GSH)_2_, at low concentrations (≤5 µM) induced autophagy in HepG2 cells as early as 12 h post-treatment and the levels of LC3-I and LC3-II were subsequently decreased at longer incubation times, resulting in the recovery and partial rescue of the cells from the stress. On the other hand, the treatment of HepG2 cells with EF25-(GSH)_2_ at higher concentrations (≥10 µM) blocked autophagy flux and led to the activation of apoptosis via caspase-dependent and caspase-independent pathways^44^. Therefore, the connection between apoptosis and autophagy should be further investigated to gain insights in the mechanisms of the programmed-cell death induction mediated by the BF of Cur and CurDD.

In summary, CurDD is an ester prodrug of Cur that enhances the anti-proliferation activity against HepG2 cells of Cur. It may exhibit anti-tumor activity against HepG2 cells by inducing apoptotic pathway. The enhanced anti-proliferative effect could be derived from the higher cellular permeation, which in turn might be derived from improving the metabolic stability of Cur. The results suggest that CurDD is a promising prodrug of Cur and has the potential to be further developed as a therapeutic agent or an adjuvant for the treatment of hepatocellular carcinoma.

## Supplementary information

Supplementary information_Curcumin diethyl disuccinate, a prodrug of curcumin, enhances anti-proliferative effect of curcumin against HepG2 cells via apoptosis induction
