# Supplementary material for: Curcumin diethyl disuccinate, a prodrug of curcumin, enhances anti-proliferative effect of curcumin against HepG2 cells via apoptosis induction
Source: Sci Rep. 2019 Aug 12;9:11718. doi: 10.1038/s41598-019-48124-1 (PMC6690956; doi:10.1038/s41598-019-48124-1)
Supplement: Supplementary file 1 — Supplementary information_Curcumin diethyl disuccinate, a prodrug of curcumin, enhances anti-proliferative effect of curcumin against HepG2 cells via apoptosis induction [file 41598_2019_48124_MOESM1_ESM.doc]

**Supplementary information**

**Curcumin diethyl disuccinate, a prodrug of curcumin, enhances anti-proliferative effect of curcumin against HepG2 cells via apoptosis induction**

Chawanphat Muangnoi^1,2^, Pahweenvaj Ratnatilaka Na Bhuket^2^, Ponsiree Jithavech^2^, Wiwat Supasena^2^, Luminita Paraoan^3^, Suthiluk Patumraj^4^ and Pornchai Rojsitthisak^2,5,*^

^1^Pharmaceutical Chemistry and Natural Products Program, Faculty of Pharmaceutical Sciences, Chulalongkorn University, Bangkok, 10330, Thailand.

^2^Natural Products for Ageing and Chronic Diseases Research Unit,

Chulalongkorn University, Bangkok, 10330, Thailand

^3^Institute of Ageing and Chronic Disease, University of Liverpool, Liverpool, UK

^4^Center of Excellence for Microcirculation, Department of Physiology, Faculty of Medicine, Chulalongkorn University, Bangkok, 10300, Thailand.

^5^Department of Food and Pharmaceutical Chemistry, Faculty of Pharmaceutical Sciences,

Chulalongkorn University, Bangkok, 10330, Thailand.

*To whom correspondence should be addressed.

Pornchai Rojsitthisak, Ph.D.

Department of Food and Pharmaceutical Chemistry,

Faculty of Pharmaceutical Sciences, Chulalongkorn University,

254 Phayathai Road, Patumwan, Bangkok 10330 Thailand

Tel.: +66-2-218-8310; Fax: +66-2-254-5195; E-mail: pornchai.r@chula.ac.th (P. Rojsitthisak)

**Evaluation of cleaved caspase-3, cleaved caspase-9, Bax, Bcl-2 and LC3B expressions**

Equal amounts (40 µg) of each protein sample (cell lysate) were applied to 10% SDS-PAGE gels. Each gel was loaded with protein samples for determination of individual protein markers. Gels were transferred to a pure nitrocellulose membrane (Amersham™Protran®, Sigma Aldrich), and blocked with 5% dry milk. The membrane was incubated with primary antibodies against cleaved caspase-3 (1:1000), cleaved caspase-9 (1:1000) Bax (1:1000), Bcl-2 (1:1,000), LC3B (1:1000) or β-actin (1:5,000) at 4°C overnight. Then, membranes were washed with TBST and incubated with species-speciﬁc HRP conjugated secondary antibody reacted with Super Signal solution (Endogen Inc, Rockford, IL, USA) for 2 min. The membrane was exposed to an X-ray ﬁlm, stripped off the bound antibody and re-probed with anti-β actin antibody to conﬁrm the equal loading of protein. The density of target bands was quantiﬁed with the Image J program (downloaded from http://rsb.info.nih.gov/ij/). Results were expressed as a relative ratio of band intensity of the target proteins and β-actin.

**Cleaved caspase-3 protein**

Lane 1 2 3

**
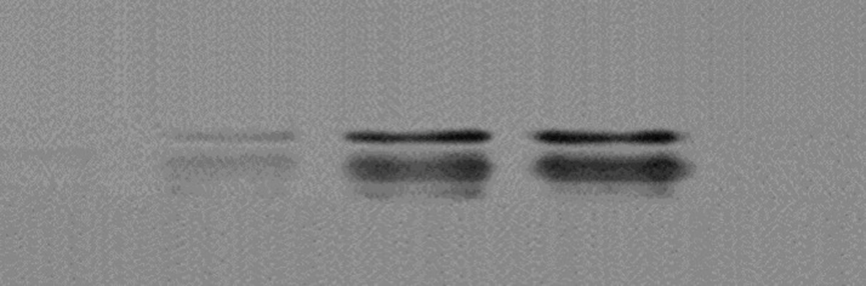
**

**β-actin for cleaved caspase-3**

Lane 1 2 3

**
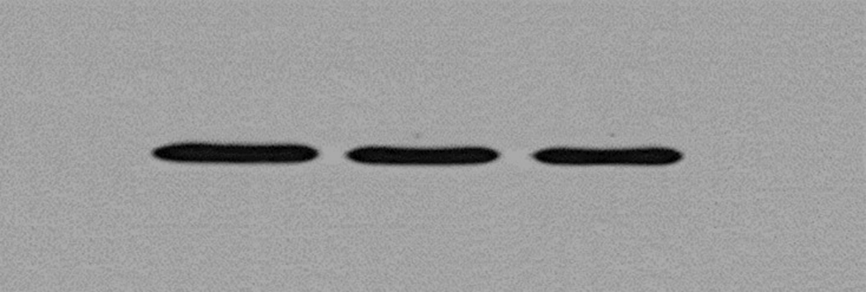
**

Lane 1; Control (Bioavailable fraction without sample)

Lane 2; Bioavailable fraction of curcumin (BF of Cur)

Lane 3; Bioavailable fraction of curcumin diethyl disuccinate (BF of CurDD)

**Cleaved caspase-9 protein**

Lane 1 2 3

**
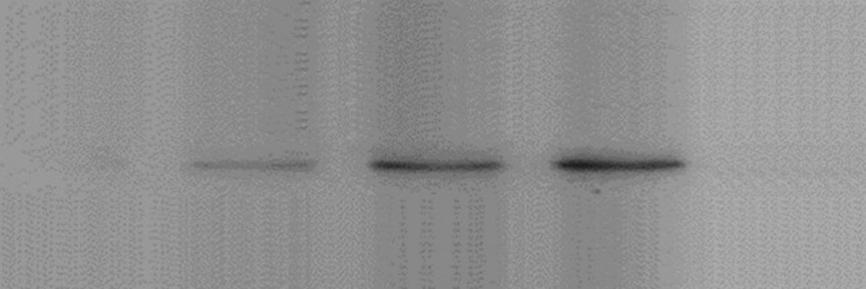
**

**β-actin for cleaved caspase-3**

Lane 1 2 3

**
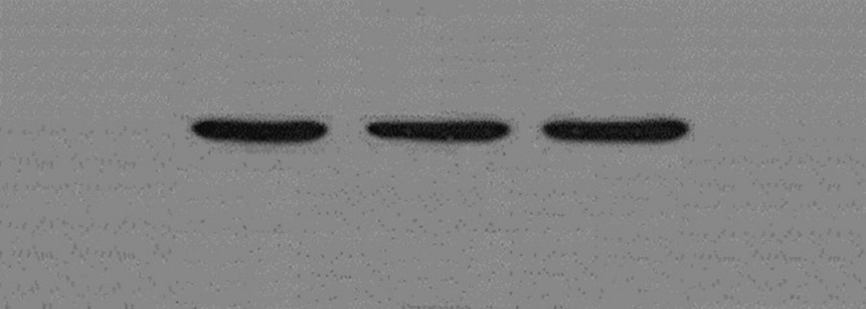
**

Lane 1; Control (Bioavailable fraction without sample)

Lane 2; Bioavailable fraction of curcumin (BF of Cur)

Lane 3; Bioavailable fraction of curcumin diethyl disuccinate (BF of CurDD)

**Bax protein**

Lane 1 2 3

**
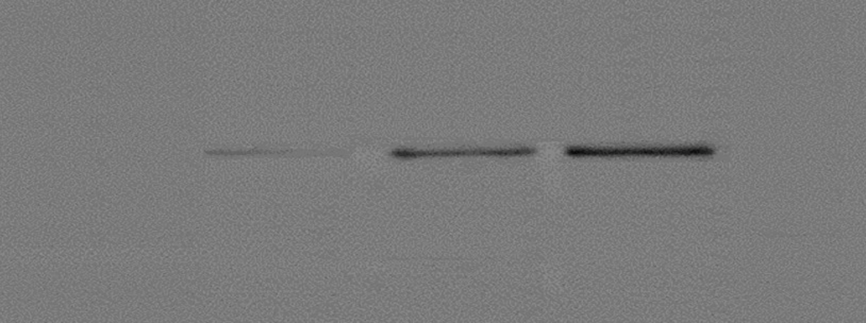
**

**β-actin for Bax**

Lane 1 2 3

**
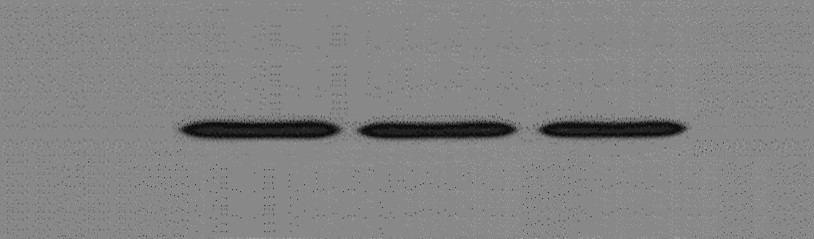
**

Lane 1; Control (Bioavailable fraction without sample)

Lane 2; Bioavailable fraction of curcumin (BF of Cur)

Lane 3; Bioavailable fraction of curcumin diethyl disuccinate (BF of CurDD)

**Bcl-2 protein**

Lane 1 2 3

**
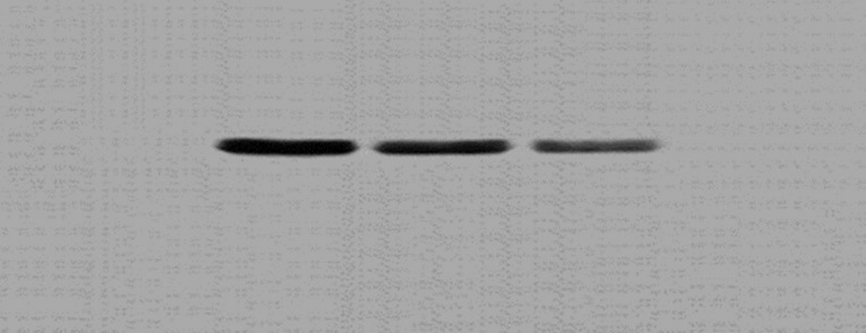
**

**β-actin for Bcl-2**

Lane 1 2 3

**
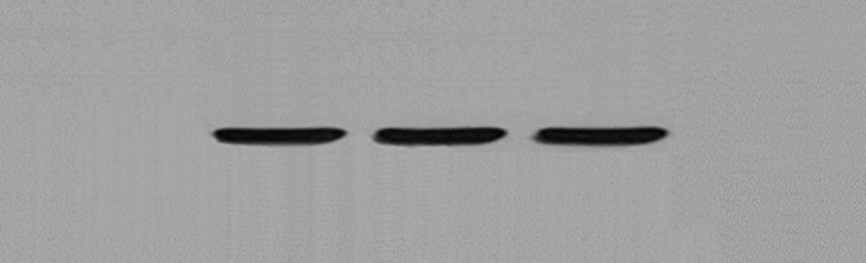
**

Lane 1; Control (Bioavailable fraction without sample)

Lane 2; Bioavailable fraction of curcumin (BF of Cur)

Lane 3; Bioavailable fraction of curcumin diethyl disuccinate (BF of CurDD)

**LC3-II protein**

Lane 1 2 3


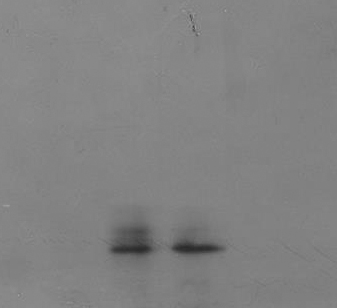


**β-actin for LC3-II**

Lane 1 2 3


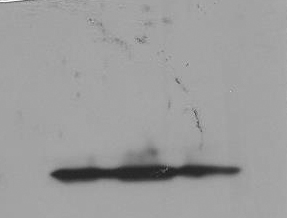


Lane 1; Control (Bioavailable fraction without sample)

Lane 2; Bioavailable fraction of curcumin (BF of Cur)

Lane 3; Bioavailable fraction of curcumin diethyl disuccinate (BF of CurDD)
